# Supplementary material for: AffyMAPSDetector: a software tool to characterize Affymetrix GeneChip™ expression arrays with respect to SNPs
Source: BMC Bioinformatics. 2007 Jul 30;8:276. doi: 10.1186/1471-2105-8-276 (PMC1959249; doi:10.1186/1471-2105-8-276)
Supplement: Additional file 13 — AffyMAPSDetector v1 source code. AffyMAPSDetector source code. [file 1471-2105-8-276-S13.zip › AffyMAPSDetector_v1_src/doc/jwf/class-use/WizardContext.html]

Uses of Class jwf.WizardContext


|  |  |  |  |
| --- | --- | --- | --- |
| **Overview** \| **Package** \| **Class** \| **Use** \| **Tree** \| **Deprecated** \| **Index** \| **Help** | | |  |
| PREV   NEXT | **FRAMES**    **NO FRAMES**     **All Classes** |  |  |


---


## **Uses of Class jwf.WizardContext**

|  |  |
| --- | --- |
| Packages that use WizardContext | |
| **jwf** | This package contains classes that can be used to create and run a Wizard. |

Uses of WizardContext in jwf

|  |  |
| --- | --- |
| Fields in jwf declared as WizardContext | |
| `protected  WizardContext` | `WizardPanel.wizardContext`             The context of the wizard process. |

|  |  |
| --- | --- |
| Methods in jwf that return WizardContext | |
| `WizardContext` | `WizardPanel.getWizardContext()`             Get the wizard context. |

|  |  |
| --- | --- |
| Methods in jwf with parameters of type WizardContext | |
| `protected  void` | `WizardPanel.setWizardContext(WizardContext wizardContext)`             Sets the context this wizard should use. |

---


|  |  |  |  |
| --- | --- | --- | --- |
| **Overview** \| **Package** \| **Class** \| **Use** \| **Tree** \| **Deprecated** \| **Index** \| **Help** | | |  |
| PREV   NEXT | **FRAMES**    **NO FRAMES**     **All Classes** |  |  |


---
